# Convenient Synthesis and Biological Evaluation of Modafinil Derivatives: Benzhydrylsulfanyl and Benzhydrylsulfinyl [1,2,3]triazol-4-yl-methyl Esters

**DOI:** 10.3390/molecules161210409

**Published:** 2011-12-15

**Authors:** Jae-Chul Jung, Yeonju Lee, Jee-Young Son, Eunyoung Lim, Mankil Jung, Seikwan Oh

**Affiliations:** 1 Department of Neuroscience and TIDRC, School of Medicine, Ewha Womans University, Seoul 158-710, Korea; 2 Department of Chemistry, Yonsei University, Seoul 120-749, Korea

**Keywords:** benzhydrylsulfanyl-[1,2,3]triazol-4-yl-methyl ester, benzhydrylsulfinyl-1,2,3]triazol-4-yl-methyl ester, NO-generation, condensation, 1,3-dipolar cycloaddition reaction

## Abstract

Simple synthesis and biological activities of modafinil derivatives are described. The key reactions include condensation of acid and propargyl alcohol, subsequent 1,3-dipolar cycloaddition reaction of alkynes and (3-azido-propyl)cyclohexane or (4-azido-butyl)benzene in the presence of sodium ascorbate and CuSO_4_·5H_2_O in excellent yield. They were then evaluated for the suppression of LPS-induced NO generation *in vitro*. It was found that all compounds showed moderate effects for suppression of LPS-induced NO generation.

## 1. Introduction

**Figure 1:**
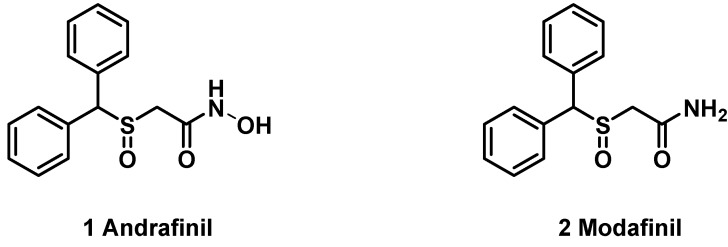
Structures of andrafinil **1** and modafinil **2**.

Modafinil [(±)-2-[di(phenyl)methylsulfinyl]acetamide] was known clinically useful in the treatment of narcolepsy, a neurological disorder marked by uncontrollable attacks of daytime sleepiness. Narcolepsy is caused by dysfunction of a family of wakefulness-promoting and sleep-suppressing peptides, the orexins. Even though the mode of action of modafinil is not been known it seems to be wake promoting activity similar to sympathomimetic agents such as amphetamine or methylphenidate. Modafinil is a structural racemic mixture including sulfinyl and amide functional groups. The (*R*)-enantiomer is known as armodafinil (Nuvigil) and *N*-oxim amide type of modafinil is andrafinil **1** as a mild central nervous system stimulant (Figure 1).

Since modafinil has been used as a psychostimulant for the treatment of narcolepsy, most research on the action mechanism of modafinil has focused on monoaminergic effects showing that modafinil stimulates the dopamine, serotonin, and norepinephrine pathways in the brain. In addition, modafinil is known to inhibit hepatic cytochrome P450 activities and has a neuroprotective function [1,2,3]. Recent reports described a simple synthesis of racemic or single isomeric modafinil and its derivatives including their screening assay for their biological properties [4,5]. It might also be useful to treat narcolepsy, attention deficit hyperactivity disorder (ADHD), and cancer-related fatigue [6,7,8]. Even though the CNS stimulants were limited to treat narcolepsy and ADHD due to serious side effects, the therapeutic usefulness and these medication effectiveness were dramatically increased for the CNS diseases [9]. It is experimentally used in the treatment of Alzheimer's disease, myotonic dystrophy, multiple sclerosis-induced fatigue, jet-lag, and cognitive impairment in schizophrenia [10,11,12]. In recent, further therapeutic potential of modafinil includes multiple sclerosis [13], Parkinson’s disease [14], attention-deficit disorder [15] and cocaine dependence and withdrawal [16]. These results led us to investigate the pharmacological activity of modafinil derivatives on the inflammation.

Recently, the Olivo group [17] has developed a new protocol for the synthesis of (+)-modafinil through microbial oxidation and amidation of benzhydrylsulfanyl acetic acid. This highly enantioselective synthesis was achieved employing the fungus *Beauveria bassiana* in 89% yield with 99% *ee*. The Prisinzano group [18] reported asymmetric synthesis of modafinil using oxidation, hydrolysis, and resolution from benzhydrol as a starting material. They also determined for the absolute configuration of enantiomers of it through X-ray crystallographic analysis. The Minzenberg group [19] comprehensively reviewed neuorochemical actions of modafinil, and effects on cognition in animal models healthy adult humans, and clinical populations.

In a continuation of our medicinal chemistry program connected with the synthesis of modafinil derivatives and evaluation of their biological activities, we required key fragment in order to generate novel anti-inflammatory analogues. We wish to describe efficient synthesis of modafinil derivatives such as benzhydrylsulfanyl or benzhydrylsulfinyl [1,2,3]triazol-4-yl-methyl esters starting from easily prepared benzhydrylsulfanyl acetic acid or 2-(benzhydrylsulfinyl)acetic acid through oxidation of sulfanyl moieties with 30%-H_2_O_2_, concd-H_2_SO_4_ or Lawesson’s reagent, condensation of acids with alcohol, and 1,3-dipolar cycloaddition reaction of alkynes with azido agents using sodium ascorbate, and CuSO_4_·5H_2_O in excellent yields.

## 2. Results and Discussion

### 2.1. Chemistry

**Scheme 1:**
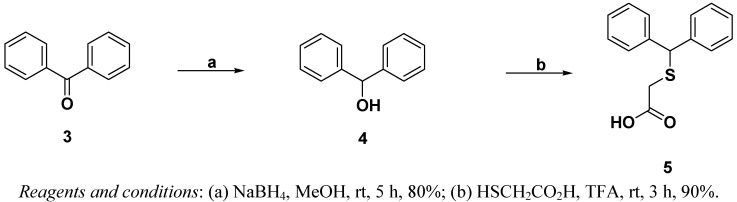
Synthesis of benzhydrylsulfanyl acetic acid.

According to continuous modafinil study, we need multigram quantities of benzhydrylsulfanyl acetic acid (**5**) for derivatization of modafinil-azido moiety to evaluate of their biological activity. The synthesis of key fragment **5** was accomplished as depicted in Scheme 1. Commercially available **3** underwent reduction with sodium hydride (60% dispersion mineral oil in hexane) in MeOH to give secondary alcohol **4** in 80% yield, which was smoothly transformed by thioglycolic acid in the presence of trifluoroacetic acid (TFA) at room temperature to generate acid **5** in 90% yield (Scheme 1).

**Scheme 2:**
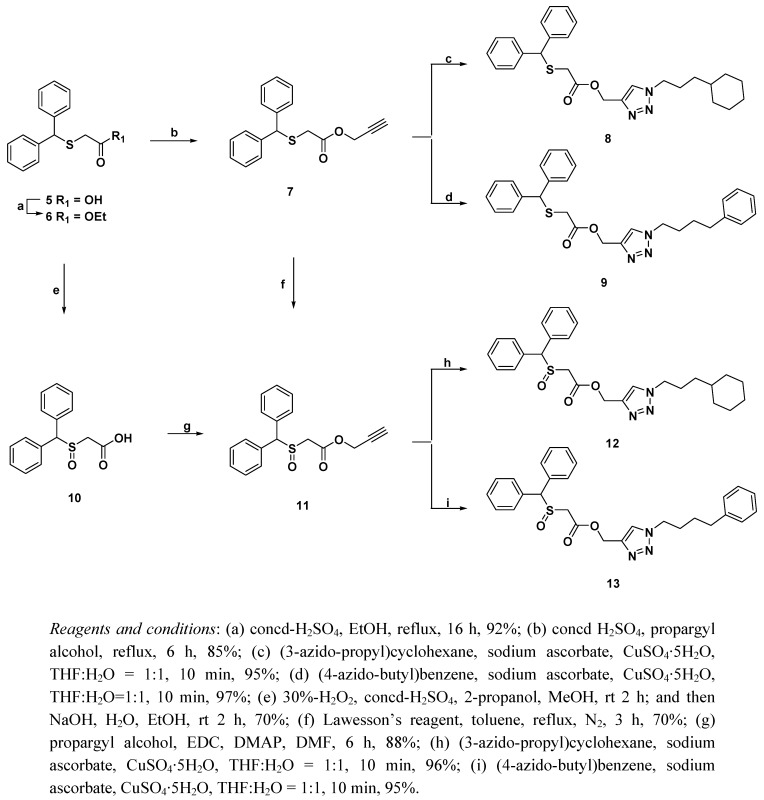
Synthesis of modafinil derivatives.

In our hands this required the intermediate acid **5** to be made in order to generate novel modafinil derivatives. Esterification of acid **5** was accomplished by using concentrated sulfuric acid in ethanol to afford ethyl ester **6** in 92% yield [20]. Also acid **6** was directly condensed with propargyl alcohol in the presence of concentrated sulfuric acid to give terminal alkyne **7** in 85% yield [21], which was readily treated with (3-azido-propyl)cyclohexane, sodium ascorbate and CuSO_4_·5H_2_O in THF/H_2_O (v/v, 1:1) or (4-azido-butyl)benzene, sodium ascorbate and CuSO_4_·5H_2_O in THF/H_2_O (v/v, 1:1) to generate compounds **8** and **9** in 95% and 97% yields, respectively [22]. Likewise, oxidation of the ethyl ester **6** performed with 30%-H_2_O_2_in acidic media in alcohol solvent which was hydrolyzed in situ under NaOH in H_2_O and EtOH to give **10** in 70% yield. Treatment of acid **10** with propargyl alcohol and ethyl(dimethylaminopropyl)carbodiimide (EDC) in the presence of 4-(dimethylamino)pyridine (DMAP) in *N*,*N*-dimethylformamide (DMF) to give **11** in 88% yield, which was then treated with (3-azido-propyl)cyclohexane, sodium ascorbate and CuSO_4_·5H_2_O in THF/H_2_O (v/v, 1:1) or (4-azido-butyl)benzene, sodium ascorbate and CuSO_4_·5H_2_O in THF/H_2_O (v/v, 1:1) for 10 min, to generate compounds **12** and **13** in 96% and 95% yields, respectively. These triazole formation via Cu (I) catalyzed 1,3-dipolar cycloaddition reaction (1,3 DCR) of azido moieties and terminal alkyne proceeded smoothly cases and under very mild conditions [23]. Unfortunately, condensation of acid **10** with propargyl alcohol in the presence of concentrated sulfuric acid did not result in the formation of compound **11**, this reaction condition was resulted in mainly detected staring material and/or decomposed product. In attempt to generate compound **11** through oxidation of compound **7** with Lawesson’s reagent in toluene was successfully prepared desired product in 70% yield (Scheme 2). The synthetic resultant the benzhydrylsulfanyl or benzhydrylsulfinyl [1,2,3]triazol-4-yl-methyl esters **8-9** and **12-13** were then evaluated for biological efficacies in *In vitro*.

### 2.2. Biology

**Figure 2:**
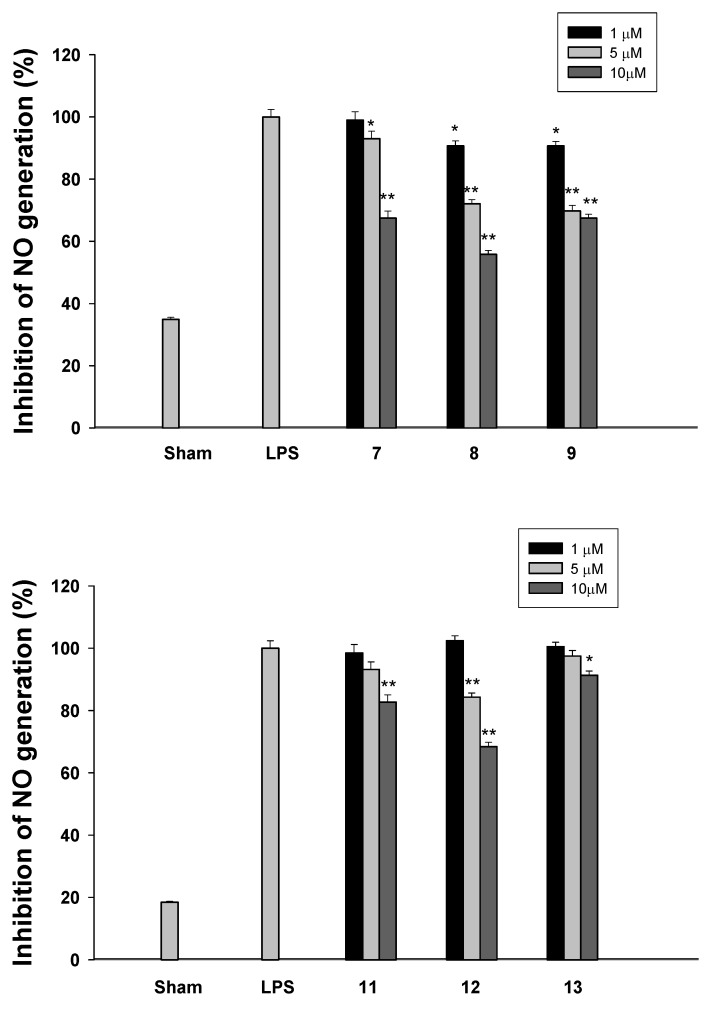
Effect of modafinil derivatives **7-9**and **11-13**on NO generation in LPS-stimulated BV-2 microglia cells. Cells were treated with 100 ng/mL LPS, and then various concentrations of these compounds (1 µM, 5 µM, and 10 µM) were added for 24 h at 37 °C. Values indicate inhibition of NO production from culture supernatants of LPS-treated cells with or without compounds. Data represent the mean ± standard deviation of three observations. * < 0.05, ** < 0.01 indicate significant difference compare with LPS alone group.

Nitrite was used as a measure of NO production. The *In vitro* suppression of LPS-induced NO generation with the prepared modafinil derivatives was evaluated by the published test method [24] and the results are summarized in Figure 2. Most of the benzhydrylsulfanyl-[1,2,3]-triazol-4-yl-methyl esters **7-9** or benzhydrylsulfinyl-[1,2,3]triazol-4-yl-methyl esters **11-13** inhibited nitrite accumulation in LPS-stimulated microglia BV-2 cells, with compound **9** exhibiting the highest inhibitory activity for LPS-induced NO generation.

We have found that the modafinil derivatives **7-9** and **11-13** exhibited potent anti-inflammatory. On the structural characteristics of the modafinil derivatives revealed that some of them are important difference for the biological activities. In case of the benzhydrylsulfanyl-[1,2,3]-triazol-4-yl-methyl esters **7-9** sulfide analogues superior anti-inflammatory activity compared to benzhydrylsulfinyl-[1,2,3]triazol-4-yl-methyl esters **11-13** (Figure 2). Interestingly, the analogues introduction of (3-azido-propyl)cyclohexane **8** and **12** showed more favorable activity than the compounds **9** and **13**. It seems to be binding affinity effect on the active sites.

## 3. Experimental Section

Reactions requiring anhydrous conditions were performed with the usual precautions for rigorous exclusion of air and moisture. Tetrahydrofuran was distilled from sodium benzophenone ketyl prior to use. Thin layer chromatography (TLC) was performed on precoated silica gel G and GP uniplates from Analtech and visualized with 254-nm UV light. Flash chromatography was carried out on silica gel 60 [Scientific Adsorbents Incorporated (SAI), particle size 32-63 µm, pore size 60 Å]. ^1^H-NMR, and ^13^C-NMR spectra were recorded on a Bruker DPX 500 at 500 MHz and 125 MHz; respectively. The chemical shifts are reported in parts per million (ppm) downfield from tetramethylsilane, and *J*-values are in Hz. Infrared (IR) spectra were obtained on an ATI Mattson FT/IR spectrometer. Mass spectra were recorded with a Waters Micromass ZQ LC-Mass system and high resolution mass spectra (HRMS) were measured with a Bruker BioApex FTMS system by direct injection using an electrospray interface (ESI). When necessary, chemicals were purified according to the reported procedures [25].

*Diphenylmethanol* (**4**). To a stirred solution of benzophenone **3** (1.25 g, 6.9 mmol) and sodium borohyde (0.75 g, 18.3 mmol) in methanol (12 mL) was stirred at room temperature 5 h. After cooling to room temperature, the reaction mixture was poured into water (15 mL) and extracted with CH_2_Cl_2_ (2 × 30 mL), washed with 5%-NaHCO_3_ (30 mL) and brine (30 mL). The organic layer was dried over anhydrous MgSO_4_, filtered and concentrated under reduced pressure. The product was purified by recrystalization by dichloromethane to give **4** (1.0 g, 80%). C_1__3_H_1__2_O_1_: White solid. mp 66.5 °C; IR_max_ (CHCl_3_, KBr) 3345, 3152, 2933, 1609, 1483, 1422, 1289, 1033, 873 cm^−1^; ^1^H-NMR (250 MHz, DMSO-d_6_) δ 7.45-7.23 (m, 10H), 5.84 (d, *J* = 3.5 Hz, 1H), 2.28 (d, *J* = 3.6 Hz, 1H); ^13^C-NMR (63 MHz, DMSO-d_6_) δ 143.5, 128.6, 127.4, 126.6, 76.3; LC-MS (ESI+) *m/z* 207-[M+Na].

*Benzhydrylsulfanyl acetic acid* (**5**). To a stirred solution of benzyhydrol **4** (1.0 g, 5.4 mmol), thioglycolic acid (0.5 g, 5.4 mmol) in TFA (6 mL) was added at room temperature and the mixture was stirred for 3 h. The solvent was removed by evaporation, and then recrystalized by water. The resulting solid was washed with *n*-hexane to yield **5** (1.26g, 90%). C_15_H_14_O_2_S: White solid. mp 130-131 °C; R*_f_* = 0.3 (*n*-Hexane/EtOAc = 1:2, v/v); IR_max_ (CHCl_3_, KBr) 3395, 3175, 2923, 1708, 1600, 1493, 1446, 1384, 1284, 1025, 750, 702, 586, 503 cm^−1^; ^1^H-NMR (250 MHz, DMSO-d_6_) δ 7.42-7.46 (m, 4H), 7.31-7.37 (m, 4H), 7.21-7.27 (m, 2H), 5.39 (s, 1H), 3.07 (s, 2H); ^13^C-NMR (63 MHz, DMSO-d_6_) δ 170.9, 140.9, 128.7, 128.0, 127.4, 53.0, 33.8; LC-MS (ESI+) *m/z* 281-[M+Na].

*2-(Benzhydrylsulfinyl)acetic acid* (**10**). Compound **7** (204 mg, 0.72 mmol) was dissolved in methanol. To a stirred solution 30%-H_2_O_2_ (0.065 mL, 2.15 mmol), acid catalyst [0.2 mL (2-propanol, 0.24 g + concd-H_2_SO_4_, 0.01 g)] was added at room temperature and the mixture was stirred for overnight. The reaction mixture was added solid NaCl (0.54 g), extracted with CH_2_Cl_2_ (3 × 50 mL) and washed with brine (2 × 80 mL). The organic layer was dried over anhydrous MgSO_4_, filtered and concentrated under reduced pressure. The crude solid was dissolved in ethanol:water = 8:1 (3.0 mL), and then NaOH (90 mg) was added and the mixture was stirred for 1 h. The solvent was removed by evaporation and then the residue was diluted with H_2_O (200 mL) which was washed with diethyl ether (2 × 50 mL). The water layer was acidified with concd-HCl to reach pH 2, and the resulting solid was filtered to give **10** (137 mg, 71%). C_15_H_14_O_3_S; White solid, mp 148-149 °C: IR_max_ (CHCl_3_, KBr) 3845, 3741, 2345, 1720, 1535, 1060, 825 cm^−1^; ^1^H-NMR (250 MHz, DMSO-d_6_) δ 13.2 (brs, 1H), 7.31-7.50 (m, 10H), 5.40 (s, 1H), 3.58 (d, *J* = 14.2 Hz, 1H), 3.33 (d, *J* = 14.2 Hz, 1H); ^13^C-NMR (63 MHz, DMSO-d_6_) δ 168.0, 137.3, 135.5, 130.3, 129.8, 129.2, 129.1, 128.8, 128.7, 69.9, 56.1; MALDI-TOF-MS: 297.03 ([M+Na]+, calcd. for C_15_H_14_O_3_S: 274.33).

### 3.1. General Experimental Procedure for Preparation of Compounds **7** and **11**

To a stirred solution of acids (**5** or **10**; 0.39 mmol) and concd-H_2_SO_4_ (0.014 mL) in propagyl alchohol (3.0 mL) was added to the reaction mixture and refluxed for 6 h. The reaction mixture was cooled to room temperature. The mixture was extracted with diethyl ether (30 mL) and washed with saturated NaHCO_3_(30 mL). The organic layer was dried over anhydrous MgSO_4_, filtered and concentrated under reduced pressure. The product was purified by flash column chromatography on silica gel (*n*-Hexane:EtOAc = 10:1, v/v) to give alkynes **7** and **11**.

*Benzhydrylsulfanyl-acetic Acid Prop-2-ynyl Ester* (**7**). C_18_H_16_O_2_S: Yield: 85%. Light yellow solid. mp 53 °C; R*_f_* = 0.50 (*n*-Hexane/EtOAc = 2:1, v/v); IR_max_ (CHCl_3_, KBr) 3298, 3027, 1736, 1599, 1494, 1450, 1274, 1124, 1027, 998, 752, 702, 631, 587 cm^−1^; ^1^H-NMR (250 MHz, CDCl_3_) δ 7.44-7.41 (m, 4H), 7.31-7.16 (m, 6H), 5.42 (s, 1H), 4.61(s, 2H), 3.07 (s, 2H), 2.48 (s, 1H); ^13^C-NMR (63 MHz, CDCl_3_) δ 169.4, 140.2, 128.7, 128.5, 127.6, 76.8, 75.5, 54.1, 52.7, 33.3; LC-MS (ESI+) *m/z* 319-[M+Na]. HRMS calcd. for C_18_H_1__6_NaO_2_S: 319.0769 [M+Na]^+^, found: 319.0881.

*2-(Benzhydrylsulfinyl)acetic acid prop-2-ynyl ester *(**11**).C_18_H_16_O_3_S: Yield: 88%. White solid. mp 82 °C; R*_f_* = 0.08 (*n*-Hexane/EtOAc = 2:1, v/v); IR_max_ (CHCl_3_, KBr) 3466, 3300, 3005, 2129, 1742, 1496, 1451, 1384, 1280, 1117, 1055, 995, 754, 703, 631, 496 cm^−1^; ^1^H-NMR (250 MHz, CDCl_3_) δ 7.51-7.45 (m, 4H), 7.40-7.31 (m, 6H), 5.22 (s, 1H), 4.70 (s, 2H), 3.54 (d, *J *= 12.5 Hz, 1H), 3.43 (d, *J* = 15.0 Hz, 1H), 2.53 (s, 1H); ^13^C-NMR (63 MHz, CDCl_3_) δ 164.7, 135.2, 129.7, 129.4, 128.9, 128.7, 76.7, 76.0, 53.9, 53.2, 32.9; LC-MS (ESI+) *m/z* 335-[M+Na]. HRMS calcd. for C_18_H_1__6_NaO_3_S: 335.0728 [M+Na]^+^, found: 335.0893.

### 3.2. General Experimental Procedure for Preparation of Compounds **8**, **9**, **12**, and **13**

To a stirred solution of ester (**7** or **11**; 0.1 mmol), appropriate azide (0.1 mmol), CuSO_4_·5H_2_O (2.5 mg, 0.01 mmol) and sodium ascorbate (4.0 mg, 0.02 mmol) in THF:H_2_O (1:1, 2.0 mL) was added at room temperature and the mixture was stirred for 10 min at same temperature. The reaction mixture was diluted with ethyl acetate (20 mL) and washed with brine (20 mL), respectively. The organic layer was separated, dried over anhydrous MgSO_4_, filtered and concentrated under reduced pressure. The product was purified by flash column chromatography on silica gel (*n*-Hexane:EtOAc = 2:1, v/v) to give compounds **8**, **9**, **12**, and **13**.

*Benzhydrylsulfanyl-acetic acid 1-{(3-cyclohexyl-propyl)-1H-[1,2,3]triazol-4-yl}methyl ester* (**8**). C_27_H_33_N_3_O_2_S: Yield: 95%. Whtie solid. mp 68.9 °C; R*_f_* = 0.27 (*n*-Hexane/EtOAc = 2:1, v/v); IR_max_ (CHCl_3_, KBr) 2922, 2850, 1736, 1493, 1449, 1269, 1143, 1125, 1050, 1030, 970, 750, 703 cm^−1^; ^1^H-NMR (250 MHz, CDCl_3_) δ 7.58 (s, 1H), 7.41-7.19 (m, 10H), 5.35 (s, 1H), 5.23 (s, 2H), 4.29 (t, *J *= 7.42 Hz, 2H), 3.08 (s, 2H), 1.94-1.82 (m, 2H), 1.67 (d, *J* = 7.50 Hz, 5H), 1.26-1.14 (m, 6H), 0.87 (t, *J* = 9.95 Hz, 2H); ^13^C-NMR (63 MHz, CDCl_3_) δ 170.3, 145.2, 142.5, 140.4, 128.8, 128.6, 127.7, 58.5, 54.2, 48.9, 38.4, 37.4, 33.7, 31.0, 22.6; LC-MS (ESI+) *m/z* 486-[M+Na]. HRMS calcd. for C_27_H_33_N_3_NaO_2_S: 486.2191 [M+Na]^+^, found: 486.2172.

*Benzhydrylsulfanyl-acetic acid 1-{(4-phenyl-butyl)-1H-[1,2,3]triazol-4-yl}methyl ester* (**9**). C_28_H_29_N_3_O_2_S: Yield: 97%. Whtie liquid. R*_f_* = 0.15 (*n*-Hexane/EtOAc = 2:1, v/v); IR_max_ (CHCl_3_, KBr) 2960, 2925, 1733, 1493, 1451, 1269, 1137, 1050, 1029, 750, 702 cm^−1^; ^1^H-NMR (250 MHz, CDCl_3_) δ 7.46 (s, 1H), 7.40-7.29 (m, 4H), 7.26-7.15 (m, 11H), 5.35 (s, 1H), 5.21 (s, 2H), 4.18 (t, *J* = 7.27 Hz, 2H), 3.08 (s, 2H), 2.25-2.18 (m, 2H), 1.26 (d, *J *= 17.5 Hz, 4H); ^13^C-NMR (63 MHz, CDCl_3_) δ 170.3, 145.2, 142.5, 140.4, 129.0, 128.8, 128.6, 127.7, 127.1, 126.9, 58.6, 54.2, 48.9, 38.4, 37.4, 33.7, 31.1, 22.6; LC-MS (ESI+) *m/z* 494-[M+Na]. HRMS calcd. for C_28_H_29_N_3_NaO_2_S: 494.1878 [M+Na]^+^, found: 494.1886.

*2-(Benzhydrylsulfinyl)acetic acid-1-{(3-cyclohexyl-propyl)-1H-[1,2,3]triazol-4-yl}methyl ester* (**12**). C_27_H_33_N_3_O_3_S: Yield: 96%. Whtie solid. mp 98 °C; R*_f_* = 0.19 (*n*-Hexane/EtOAc = 1:1, v/v); IR_max_ (CHCl_3_, KBr) 3440, 2922, 2850, 1738, 1495, 1450, 1384, 1282, 1153, 1117, 1053, 967, 752, 704 cm^−1^; ^1^H-NMR (250 MHz, CDCl_3_) δ 7.64 (s, 1H), 7.49-7.28 (m, 10H), 5.29 (s, 2H), 5.19 (s, 1H), 4.29 (t, *J* = 7.27 Hz, 2H), 3.54 (d, *J* = 15.0 Hz, 1H), 3.42 (d, *J* = 12.5 Hz, 1H), 1.93-1.82 (m, 2H), 1.66 (d, *J* = 7.50 Hz, 5H), 1.26-1.03 (m, 6H), 0.87 (t, *J* = 9.95 Hz, 2H); ^13^C-NMR (63 MHz, CDCl_3_) δ 165.4, 142.1, 135.3, 133.4, 130.0, 129.5, 128.9, 128.7, 123.9, 71.8, 59.2, 54.1, 50.9, 37.2, 34.1, 33.2, 27.8, 26.6, 26.3; LC-MS (ESI+) *m/z* 502-[M+Na]. HRMS calcd. for C_27_H_33_N_3_NaO_3_S: 502.2140 [M+Na]^+^, found: 502.2124.

2-(Benzhydrylsulfinyl)acetic acid-1-{(4-phenyl-butyl)-1H-[1,2,3]triazol-4-yl}methyl ester (**13**). C_28_H_29_N_3_O_3_S: Yield: 95%. Whtie liquid. R*_f_* = 0.15 (*n*-Hexane/EtOAc = 1:1, v/v); IR_nmax_ (CHCl_3_, KBr) 2961, 2926, 1736, 1494, 1451, 1282, 1053, 1031, 967, 753, 702 cm^−1^; ^1^H-NMR (250 MHz, CDCl_3_) δ 7.53 (s, 1H), 7.46-7.15 (m, 15H), 5.28 (s, 2H), 5.19 (s, 1H), 4.17 (t, *J* = 7.42 Hz, 2H), 3.54 (d, *J* = 15.0 Hz, 1H), 3.41 (d, *J *= 15.0 Hz, 1H), 2.27-2.18 (m, 2H) 1.25 (d, *J* = 17.5 Hz, 4H); ^13^C-NMR (63 MHz, CDCl_3_) δ 165.3, 145.2, 142.0, 135.2, 134.0, 129.7, 129.5, 129.0, 128.9, 128.8, 127.1, 126.8, 71.8, 59.2, 54.1, 48.9, 38.3, 37.4, 22.5; LC-MS (ESI+) *m/z* 510-[M+Na]. HRMS calcd. for C_28_H_29_N_3_NaO_3_S: 510.1827 [M+Na]^+^, found: 510.1819.

### 3.3. BV-2 Microglia Culture

The murine BV-2 microglia cell line was maintained in DMEM supplemented with 10% FBS and penicillin/streptomycin at 37 °C in a humidified incubator under 5% CO_2_. For all experiments, cells were plated at a density of 1 × 10^5^ cells/mL in 24-well plates and then treated with 100 ng/mL LPS alone or with various concentrations of compounds for 24 h at 37 °C.

### 3.4. Nitric Oxide Generation Assay

The Griess reaction was used to perform nitrite (NO metabolite) assays. Cells were incubated with LPS (lipopolysaccharide, 100 ng/mL) and various concentrations of modafinil derivatives for 24 h at 37 °C. The culture media was then mixed with an equal volume of reagent (1 part 0.1% *N*-1-naphthylethylenediamine dihydrochloride, 1 part 1% sulfanilamide in 5% phosphoric acid) in 96-well plates. The absorbance was determined at 540 nm using a microplate reader. Data are reported as the mean ± the standard deviation of three observations.

## 4. Conclusions

In conclusion, we have demonstrated a new and practical synthetic route in terms of preparation of benzhydrylsulfanyl or benzhydrylsulfinyl-[1,2,3]-triazol-4-yl-methyl esters using readily available inexpensive reagents and simple reaction conditions that do not require any special equipment or techniques. Their biological activities showed good efficacies for suppressing LPS-induced NO generation. We have found that the sulfanyl moieties **7-9** superior anti-inflammatory activity compared to sulfinyl derivatives **11-13**. These results suggest that benzhydrylsulfanyl or benzhydrylsulfinyl-[1,2,3]-triazol-4-yl-methyl esters could be useful for the development of anti-inflammatory agents.
